# Supplementary material for: Refined Analysis of Chronic White Matter Changes after Traumatic Brain Injury and Repeated Sports-Related Concussions: Of Use in Targeted Rehabilitative Approaches?
Source: J Clin Med. 2022 Jan 12;11(2):358. doi: 10.3390/jcm11020358 (PMC8780504; doi:10.3390/jcm11020358)
Supplement: Supplementary file 1 [file jcm-11-00358-s001.zip › jcm-1513942 suppl.pdf]

Supplementary Materials Table S1. Median with interquartile range for all white matter pathways and DTI-based parametric values.

White Matter Pathway	AD			FA			RD		
	HC	TBI	rSRC	HC	TBI	rSRC	HC	TBI	rSRC
AC	1.224<br>1.134–1.318	1.259<br>1.190–1.345	1.252<br>1.177–1.336	0.402<br>0.348–0.462	0.394<br>0.290–0.43	0.423<br>0.367–0.480	0.649<br>0.604–0.721	0.716<br>0.656–0.807	0.630<br>0.589–0.694
AF L	1.099<br>1.071–1.139	1.149<br>1.093–1.168	1.127<br>1.091–1.153	0.418<br>0.380–0.454	0.448<br>0.390–0.464	0.445<br>0.404–0.469	0.577<br>0.558–0.608	0.572<br>0.552–0.605	0.561<br>0.543–0.599
AF R	1.131<br>1.050–1.181	1.137<br>1.104–1.166	1.129<br>1.051–1.161	0.469<br>0.341–0.519	0.453<br>0.346–0.495	0.416<br>0.351–0.517	0.527<br>0.496–0.624	0.551<br>0.512–0.639	0.524<br>0.494–0.606
Internal capsule Anterior L	1.235<br>1.193–1.278	1.410<br>1.348–1.457	1.254<br>1.217–1.273	0.453<br>0.439–0.467	0.414<br>0.398–0.426	0.464<br>0.446–0.475	0.604<br>0.585–0.633	0.794<br>0.722–0.814	0.596<br>0.580–0.626
Internal capsule Anterior R	1.173<br>1.144–1.241	1.320<br>1.296–1.339	1.188<br>1.150–1.237	0.454<br>0.411–0.485	0.426<br>0.376–0.461	0.475<br>0.460–0.480	0.561<br>0.536–0.616	0.680<br>0.626–0.735	0.567<br>0.536–0.609
FAT L	1.140<br>1.105–1.199	1.184<br>1.123–1.234	1.145<br>1.108–1.230	0.442<br>0.362–0.515	0.419<br>0.347–0.481	0.445<br>0.377–0.525	0.565<br>0.517–0.632	0.595<br>0.553–0.672	0.563<br>0.516–0.614
FAT R	1.127<br>1.077–1.156	1.126<br>1.098–1.183	1.123<br>1.086–1.167	0.357<br>0.289–0.402	0.319<br>0.240–0.411	0.371<br>0.294–0.428	0.630<br>0.586–0.725	0.677<br>0.593–0.841	0.617<br>0.575–0.730
ATR L	1.187<br>1.102–1.274	1.234<br>1.182–1.472	1.196<br>1.137–1.277	0.419<br>0.353–0.493	0.360<br>0.313–0.440	0.439<br>0.370–0.499	0.597<br>0.553–0.642	0.709<br>0.607–0.921	0.581<br>0.538–0.653
ATR R	1.185<br>1.118–1.247	1.286<br>1.199–1.469	1.164<br>1.113–1.263	0.397<br>0.334–0.462	0.363<br>0.299–0.404	0.404<br>0.319–0.507	0.624<br>0.568–0.670	0.749<br>0.670–0.933	0.602<br>0.540–0.671
Ci L	1.147<br>1.060–1.273	1.180<br>1.086–1.258	1.186<br>1.087–1.336	0.371<br>0.291–0.414	0.323<br>0.272–0.351	0.366<br>0.299–0.431	0.652<br>0.614–0.689	0.703<br>0.673–0.778	0.651<br>0.631–0.704
Ci R	1.154<br>1.119–1.207	1.192<br>1.146–1.244	1.173<br>1.127–1.227	0.367<br>0.339–0.428	0.358<br>0.325–0.429	0.381<br>0.355–0.430	0.624<br>0.605–0.668	0.663<br>0.621–0.719	0.619<br>0.603–0.651
CS L	1.171<br>1.102–1.368	1.238<br>1.145–1.406	1.177<br>1.117–1.390	0.542<br>0.465–0.690	0.531<br>0.451–0.656	0.540<br>0.468–0.692	0.468<br>0.384–0.525	0.504<br>0.425–0.558	0.469<br>0.387–0.524
CS R	1.151<br>1.088–1.352	1.249<br>1.119–1.374	1.162<br>1.092–1.344	0.531<br>0.420–0.642	0.507<br>0.410–0.620	0.523<br>0.425–0.636	0.508<br>0.447–0.558	0.534<br>0.461–0.593	0.500<br>0.461–0.561
External capsule L	1.191<br>1.160–1.337	1.217<br>1.181–1.397	1.197<br>1.167–1.336	0.442<br>0.426–0.462	0.427<br>0.404–0.460	0.452<br>0.426–0.462	0.579<br>0.561–0.652	0.612<br>0.575–0.754	0.577<br>0.551–0.637

External capsule R	1.220<br>1.139–1.252	1.247<br>1.202–1.336	1.225<br>1.134–1.261	0.452<br>0.380–0.491	0.439<br>0.372–0.486	0.457<br>0.379–0.492	0.589<br>0.558–0.634	0.622<br>0.609–0.680	0.583<br>0.560–0.629
FM	1.536<br>1.229–1.680	1.673<br>1.331–1.811	1.564<br>1.209–1.658	0.558<br>0.363–0.696	0.536<br>0.359–0.632	0.615<br>0.394–0.717	0.581<br>0.525–0.702	0.697<br>0.652–0.778	0.518<br>0.455–0.641
Fo L	1.670<br>1.568–1.872	2.306<br>2.050–2.448	1.597<br>1.510–1.761	0.368<br>0.322–0.393	0.305<br>0.281–0.333	0.368<br>0.329–0.405	0.968<br>0.886–1.047	1.502<br>1.272–1.633	0.931<br>0.842–0.999
Fo R	1.693<br>1.593–1.902	2.416<br>1.853–2.578	1.586<br>1.501–1.849	0.315<br>0.304–0.378	0.267<br>0.248–0.308	0.328<br>0.316–0.398	1.011<br>0.868–1.229	1.615<br>1.146–1.866	0.918<br>0.831–1.159
Genu CC	1.265<br>1.180–1.481	1.296<br>1.190–1.623	1.248<br>1.156–1.483	0.448<br>0.419–0.653	0.425<br>0.382–0.615	0.460<br>0.419–0.688	0.613<br>0.490–0.633	0.670<br>0.596–0.715	0.595<br>0.438–0.618
hSLF L	1.096<br>1.083–1.116	1.128<br>1.115–1.146	1.099<br>1.081–1.119	0.390<br>0.354–0.407	0.373<br>0.341–0.393	0.386<br>0.362–0.403	0.608<br>0.585–0.645	0.659<br>0.615–0.700	0.608<br>0.581–0.645
hSLF R	1.079<br>1.076–1.085	1.114<br>1.109–1.124	1.084<br>1.075–1.095	0.381<br>0.329–0.399	0.358<br>0.318–0.374	0.384<br>0.329–0.402	0.609<br>0.589–0.669	0.659<br>0.641–0.721	0.600<br>0.582–0.675
IFOF L	1.232<br>1.164–1.459	1.270<br>1.199–1.532	1.248<br>1.192–1.460	0.454<br>0.433–0.487	0.444<br>0.400–0.482	0.464<br>0.437–0.493	0.583<br>0.556–0.651	0.619<br>0.569–0.724	0.581<br>0.542–0.634
IFOF R	1.260<br>1.146–1.302	1.281<br>1.216–1.385	1.261<br>1.136–1.315	0.480<br>0.398–0.523	0.464<br>0.389–0.533	0.487<br>0.393–0.528	0.572<br>0.540–0.616	0.618<br>0.589–0.669	0.565<br>0.543–0.618
ILF L	1.266<br>1.112–1.389	1.292<br>1.140–1.1426	1.291<br>1.137–1.389	0.454<br>0.354–0.536	0.454<br>0.351–0.505	0.478<br>0.378–0.529	0.600<br>0.548–0.634	0.642<br>0.604–0.675	0.594<br>0.553–0.630
ILF R	1.142<br>1.109–1.244	1.177<br>1.134–1.252	1.165<br>1.109–1.237	0.449<br>0.369–0.537	0.423<br>0.359–0.527	0.455<br>0.396–0.529	0.547<br>0.501–0.620	0.595<br>0.525–0.657	0.544<br>0.502–0.596
MLF L	1.250<br>1.097–1.366	1.255<br>1.105–1.382	1.301<br>1.091–1.389	0.464<br>0.285–0.537	0.453<br>0.293–0.507	0.483<br>0.315–0.553	0.590<br>0.544–0.703	0.646<br>0.587–0.711	0.597<br>0.538–0.690
MLF R	1.168<br>1.110–1.235	1.164<br>1.108–1.261	1.164<br>1.094–1.246	0.453<br>0.396–0.476	0.416<br>0.354–0.467	0.462<br>0.403–0.497	0.574<br>0.551–0.602	0.602<br>0.568–0.636	0.554<br>0.540–0.584
OR L	1.292<br>1.117–1.569	1.551<br>1.174–1.920	1.363<br>1.171–1.450	0.393<br>0.324–0.520	0.365<br>0.305–0.479	0.414<br>0.332–0.515	0.688<br>0.539–0.902	0.865<br>0.616–1.174	0.708<br>0.544–0.841
OR R	1.311<br>1.201–1.375	1.391<br>1.240–1.468	1.321<br>1.198–1.363	0.550<br>0.501–0.580	0.547<br>0.479–0.581	0.547<br>0.508–0.580	0.548<br>0.480–0.593	0.603<br>0.492–0.648	0.516<br>0.493–0.564
Internal capsule posterior L	1.191<br>1.154–1.278	1.285<br>1.190–1.333	1.206<br>1.158–1.301	0.531<br>0.481–0.587	0.506<br>0.446–0.552	0.527<br>0.477–0.585	0.500<br>0.466–0.537	0.569<br>0.531–0.594	0.499<br>0.468–0.535

Internal capsule posterior R	1.206<br>1.115–1.273	1.283<br>1.159–1.356	1.210<br>1.110–1.277	0.506<br>0.432–0.564	0.488<br>0.420–0.553	0.503<br>0.424–0.555	0.555<br>0.489–0.575	0.589<br>0.517–0.656	0.555<br>0.504–0.575
UF L	1.138<br>1.125–1.175	1.163<br>1.139–1.241	1.149<br>1.109–1.186	0.323<br>0.260–0.356	0.315<br>0.248–0.347	0.338<br>0.286–0.363	0.694<br>0.660–0.744	0.707<br>0.694–0.756	0.680<br>0.653–0.717
UF R	1.177<br>1.095–1.210	1.182<br>1.106–1.230	1.173<br>1.100–1.192	0.341<br>0.252–0.384	0.318<br>0.236–0.365	0.358<br>0.282–0.382	0.694<br>0.658–0.741	0.701<br>0.688–0.754	0.668<br>0.644–0.703
VO L	1.160<br>1.021–1.288	1.129<br>1.072–1.249	1.164<br>1.069–1.245	0.452<br>0.321–0.539	0.431<br>0.304–0.491	0.450<br>0.325–0.521	0.564<br>0.524–0.623	0.580<br>0.558–0.666	0.561<br>0.521–0.642
VO R	1.022<br>0.997–1.119	1.053<br>1.018–1.129	1.033<br>1.002–1.095	0.369<br>0.279–0.467	0.366<br>0.277–0.446	0.346<br>0.271–0.436	0.590<br>0.526–0.669	0.603<br>0.551–0.663	0.606<br>0.512–0.651
vSLF L	1.058<br>1.041–1.139	1.083<br>1.057–1.159	1.082<br>1.058–1.139	0.358<br>0.249–0.436	0.330<br>0.222–0.417	0.363<br>0.262–0.447	0.616<br>0.548–0.732	0.690<br>0.578–0.772	0.622<br>0.641–0.725
vSLF R	1.022<br>1.002–1.075	1.067<br>1.026–1.092	1.029<br>1.012–1.091	0.325<br>0.235–0.432	0.294<br>0.225–0.385	0.367<br>0.253–0.432	0.620<br>0.539–0.718	0.662<br>0.588–0.781	0.589<br>0.540–0.694

Grey shadowed cells indicate significant difference ($p < 0.05$) between TBI and HC or rSRC and HC calculated with Kruskal–Wallis test with Dunn's test to correct for multiple comparisons. The exact *p*-values can be found in Table 2.
